# Insights into the voltage-gated sodium channel, Na_V_1.8, and its role in visceral pain perception 

**DOI:** 10.3389/fphar.2024.1398409

**Published:** 2024-05-23

**Authors:** J. Westley Heinle, Shannon Dalessio, Piotr Janicki, Ann Ouyang, Kent E. Vrana, Victor Ruiz-Velasco, Matthew D. Coates

**Affiliations:** ^1^ Division of Gastroenterology and Hepatology, Penn State College of Medicine, Hershey, PA, United States; ^2^ Department of Anesthesiology and Perioperative Medicine, Penn State College of Medicine, Hershey, PA, United States; ^3^ Department of Pharmacology, Penn State College of Medicine, Hershey, PA, United States

**Keywords:** Nav1.8, pain, visceral, abdominal, voltage-gated sodium channel

## Abstract

Pain is a major issue in healthcare throughout the world. It remains one of the major clinical issues of our time because it is a common sequela of numerous conditions, has a tremendous impact on individual quality of life, and is one of the top drivers of cost in medicine, due to its influence on healthcare expenditures and lost productivity in those affected by it. Patients and healthcare providers remain desperate to find new, safer and more effective analgesics. Growing evidence indicates that the voltage-gated sodium channel Na_v_1.8 plays a critical role in transmission of pain-related signals throughout the body. For that reason, this channel appears to have strong potential to help develop novel, more selective, safer, and efficacious analgesics. However, many questions related to the physiology, function, and clinical utility of Na_v_1.8 remain to be answered. In this article, we discuss the latest studies evaluating the role of Na_v_1.8 in pain, with a particular focus on visceral pain, as well as the steps taken thus far to evaluate its potential as an analgesic target. We also review the limitations of currently available studies related to this topic, and describe the next scientific steps that have already been undertaken, or that will need to be pursued, to fully unlock the capabilities of this potential therapeutic target.

## Introduction

**FIGURE 1:**
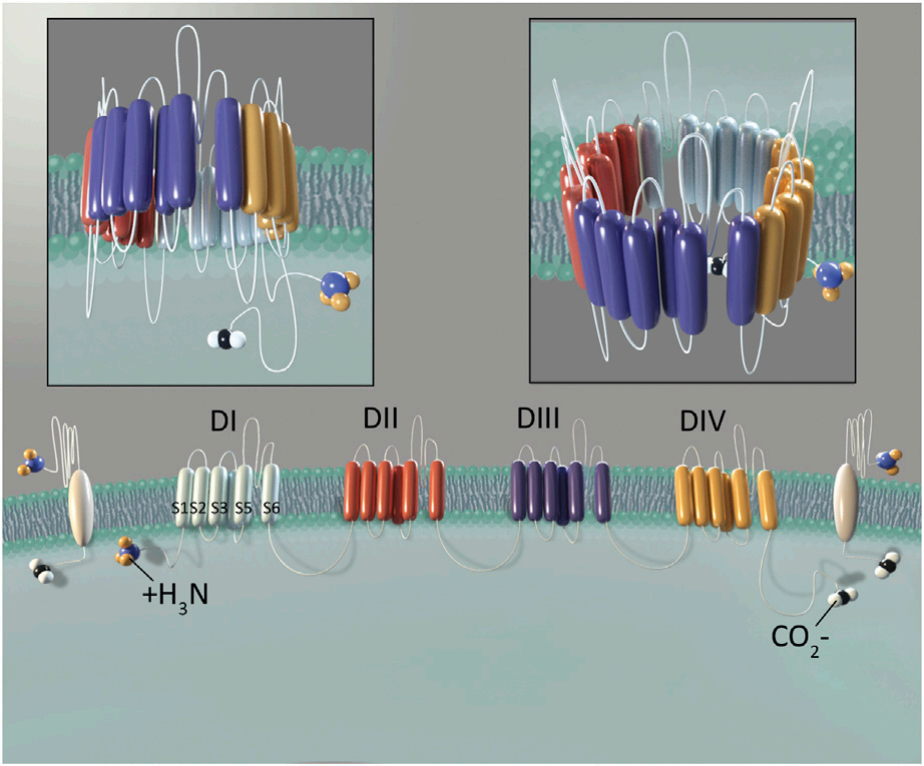
Structure of Na_V_1.8. The alpha subunit consists of four domains (DI-DIV). Domains are shown in different colors to demonstrate their independent, yet similar structure. Six transmembrane segments (S1-S6) comprise each domain. Two beta subunits are shown in white, laterally to the outside of DI and DIV. Illustration by Devon Stuart.

Voltage-gated sodium channels (VGSCs) are part of a larger family of mammalian ion channels (Catterall et al., 2005b). VGSCs are transmembrane proteins that help to regulate the membrane potential of cells. They do this by providing a hydrophilic corridor that permits controlled movement of sodium ions through the outer hydrophobic phospholipid bilayer of eukaryotic cells. To date, nine distinct VGSC isoforms have been identified in humans, named Na_v_1.1 through Na_v_1.9 (numbered based upon the order in which each type was identified) (Goldin et al., 2000; Catterall et al., 2005a). These isoforms are structurally differentiated based upon their pore-forming alpha subunits (Yu and Catterall, 2003). A VGSC is composed of one of these alpha subunits and one or more beta subunits. The alpha subunit contains six alpha-helical transmembrane segments (S1-S6) that are folded into four domains (I-IV) (Catterall, 2000). The four homologous domains of the alpha subunit form an ion-conducting aqueous pore. This region determines the specific function of each particular VGSC, including ion selectivity, expression location and overall channel function. The beta subunits influence gating and signaling functions of each channel, and also serve to help anchor these channels within a cell membrane (Yu et al., 2003; Yu and Catterall, 2004) (Figure 1).

VGSCs are encoded by the *SCN* gene family. These genes are numbered 1–11A, corresponding in sequential fashion with the isoforms described above (with the exception of SCN6-7, which encode NaX, a non-selective TTX-sensitive channel (Kanellopoulos and Matsuyama, 2016; Cheng et al., 2021; Noland et al., 2022). The product of each gene is described, in part, by the prefix Na_v_1, while the number following the decimal point identifies a specific channel isoform. Of note, these were assigned in the approximate order in which each gene was identified (Goldin et al., 2000). A more in-depth review of the structure and physiology of each VGSC can be found in Coates et al., Neurogastroenterology and Motility, 2019 (Coates et al., 2019). While the precise functions of these channels may vary, all are important determinants of the excitability of cells, including in neurons, muscles and endocrine cells. Accordingly, they play a crucial role in muscle contractions and the initiation and propagation of action potentials (Cheng et al., 2021). These channels are expressed throughout human body. Certain subtypes appear to be preferentially expressed in either the central nervous system (CNS), or peripheral nervous system (PNS), but some may appear in both (de Lera Ruiz and Kraus, 2015). For example, Nav1.4 has been chiefly associated with skeletal muscle, and genetic variations associated with its gene, *SCN4A*, have been linked to several disorders of muscular function (Mannikko et al., 2018). On the other hand, Nav1.6 appears to be expressed primarily on the membranes of neural structures within the central nervous system (Caldwell et al., 2000). Na_v_1.7, Na_v_1.8, and Na_v_1.9 are almost exclusively expressed in the peripheral nervous system (PNS) on primary sensory neurons (Bennett et al., 2019; Cheng et al., 2021). In fact, Na_v_1.8 is known to be expressed in dorsal root ganglion (DRG) and trigeminal ganglion (TG) neurons (Djouhri et al., 2003), including cells that play a major role in transmission of pain-related signals. As much as any VGSC, Na_v_1.8 has also been implicated as a significant factor in the initiation and persistence of chronic pain in a wide range of disorders, including those related to the viscera (Beyak et al., 2004; Hillsley et al., 2006; King et al., 2009; Chen et al., 2013; Hu et al., 2013; Hu et al., 2016; Lin et al., 2017). In order to understand why Na_v_1.8 is so relevant to understanding visceral pain, and represents a promising therapeutic target, it is important to review what is known about its structure and physiology, and to discuss how it has been tested in this context.

## The structure and physiology of Na_v_1.8, and its role in somatosensory pain perception

Na_v_1.8 is encoded by the gene *SCN10A* which is located on human chromosome 3p22-24 (Goldin et al., 2000). This gene has 28 exons, 27 of which are coding while one is non-coding. *SCN10A* is found in a gene cluster with *SCN5A* (which encodes Na_V_1.5) and *SCN11A* (which encodes Na_V_1.9). Notably, Na_V_1.5, Na_V_1.8 and Na_V_1.9 are all relatively resistant to the blocking effects of tetrodotoxin (TTX) when compared to other VGSCs (Kostyuk et al., 1981; Blair and Bean, 2002). This is important as TTX (a neurotoxin derived from pufferfish species) has been a critically important agent used to differentiate subtypes of VGSCs for decades (Catterall, 1980; Stevens et al., 2011).

In humans, Na_V_1.8 is composed of 1956 amino acids, and has a molecular weight of 220 kilodaltons (kDa), though this can vary in other species (Hameed, 2019). The structure of purified human Na_V_1.8 has been evaluated using cryogenic electron microscopy and there are unique aspects of the first voltage-sensing domain (VSD1) that appear to modify electrophysiological characteristics of the channel (Huang et al., 2022). Notably, compared with other VGSC isoforms, Na_V_1.8 exhibits several distinctive biophysical characteristics, including its activation at a more pronounced state of cellular depolarization, as well as slower inactivation. Na_V_1.8 remains active at voltages that inactivate other VGSCs. These features help to facilitate cellular hyperexcitability, and make Na_V_1.8 a major contributor to the depolarization phase in action potentials of nociceptive neurons. (Renganathan et al., 2000; Renganathan et al., 2001). These characteristics also help to differentiate Na_V_1.8 from Na_V_1.9, which (through the use of knockout rodent models) appears to be primarily involved with setting resting membrane potential and less important to the production of action potentials (Cummins et al., 1999; Dib-Hajj et al., 2002).

Since its discovery, Na_V_1.8 has demonstrated a strong association with nociceptors and pain perception. Na_V_1.8 was described as the “sensory neuron specific” (SNS) channel, as it was originally identified in the mucosal neurites and soma of small fiber DRG-associated neurons and vagal afferent neurons associated with pain in mammals. Akopian et al. first described the basic electrophysiological and pharmacological characteristics of Na_V_1.8, using *in situ* hybridization to determine that it was predominantly localized to small diameter neurons within the TG and DRG neurons (Akopian et al., 1996). Djouhri et al. demonstrated Na_v_1.8 immunoreactivity in small-to-medium sized DRG neurons, but not in the brain, cardiac, skeletal muscle, or a variety of other tissues (ex: liver, kidney) (Djouhri et al., 2003). Other studies performed by Akopian et al. established the importance of this channel to pain perception, when they demonstrated that mouse Na_V_1.8 knockout models exhibit pronounced analgesia to noxious mechanical and thermal stimuli, as well as delayed development of inflammatory hyperalgesia (Akopian et al., 1999). Other studies evaluating *SCN10A* genetic variants have provided further supportive evidence of the importance that Na_V_1.8 has in pain. For example, Faber et al. described the discovery of three polymorphisms they found associated with peripheral neuropathy, two of which resulted in apparent enhanced response of Na_V_1.8 channels to depolarization and the hyperexcitability of the associated neurons (L554P and A1304T) (Faber et al., 2012). This group described another polymorphism (G1662S) associated with painful small fiber neuropathy (Han et al., 2014) (Table 1).

**TABLE 1:** Clinically Relevant SCN10A (NaV1.8) Polymorphisms.

Polymorphism	Effects		References
	Animal Studies	Human Studies	
A1304T	Rodent DRG and SCG neurons transfected with the variant channel demonstrated altered electrophysiological profile (increased excitability)	Association with peripheral neuropathy	Faber et al. (2012)
L554P	Rodent DRG and SCG neurons transfected with the variant channel demonstrated altered electrophysiological profile (increased excitability)	Association with peripheral neuropathy	Faber et al. (2012)
***A2884G***		Variant associated with reduced risk for functional dyspepsia, epigastric pain syndrome and post-prandial distress syndrome	Arisawa et al. (2013)
***C3218T***		Variant associated with reduced risk for functional dyspepsia, epigastric pain syndrome and post-prandial distress syndrome	Arisawa et al. (2013)
***T3275C***		Variant associated with reduced risk for functional dyspepsia, epigastric pain syndrome and post-prandial distress syndrome	Arisawa et al. (2013)
G1662S		Variant channel was associated with impaired inactivation	Han et al. (2014)
		Carriers of the mutation exhibited painful small fiber neuropathy	
***A1073V***	Rodent DRG and SCG neurons transfected with the variant channel demonstrated altered electrophysiological profiles	*Homozygotes exhibited*	Duan et al. (2016)
		a) increased tolerance to somatosensory pain stimuli b) increased likelihood of hypoalgesic IBD, and c) reduced pain medication requirement after sigmoidectomy	Gonzalez-Lopez et al. (2018)
			Coates et al. (2019)

Note: Variants associated with alterations in visceral pain perception are noted in bold and italics.

There is also evidence that Na_V_1.8 may influence other key factors that impact nociception and pain perception. For example, in a mouse model of psoriasis, Na_V_1.8-bearing sensory neurons were found to interact with antigen presenting cells to influence the expression of particular cytokines (interleukin-12/interleukin-23) (Riol-Blanco et al., 2014), which, in turn, may affect activity of the neuron itself.

**FIGURE 2:**
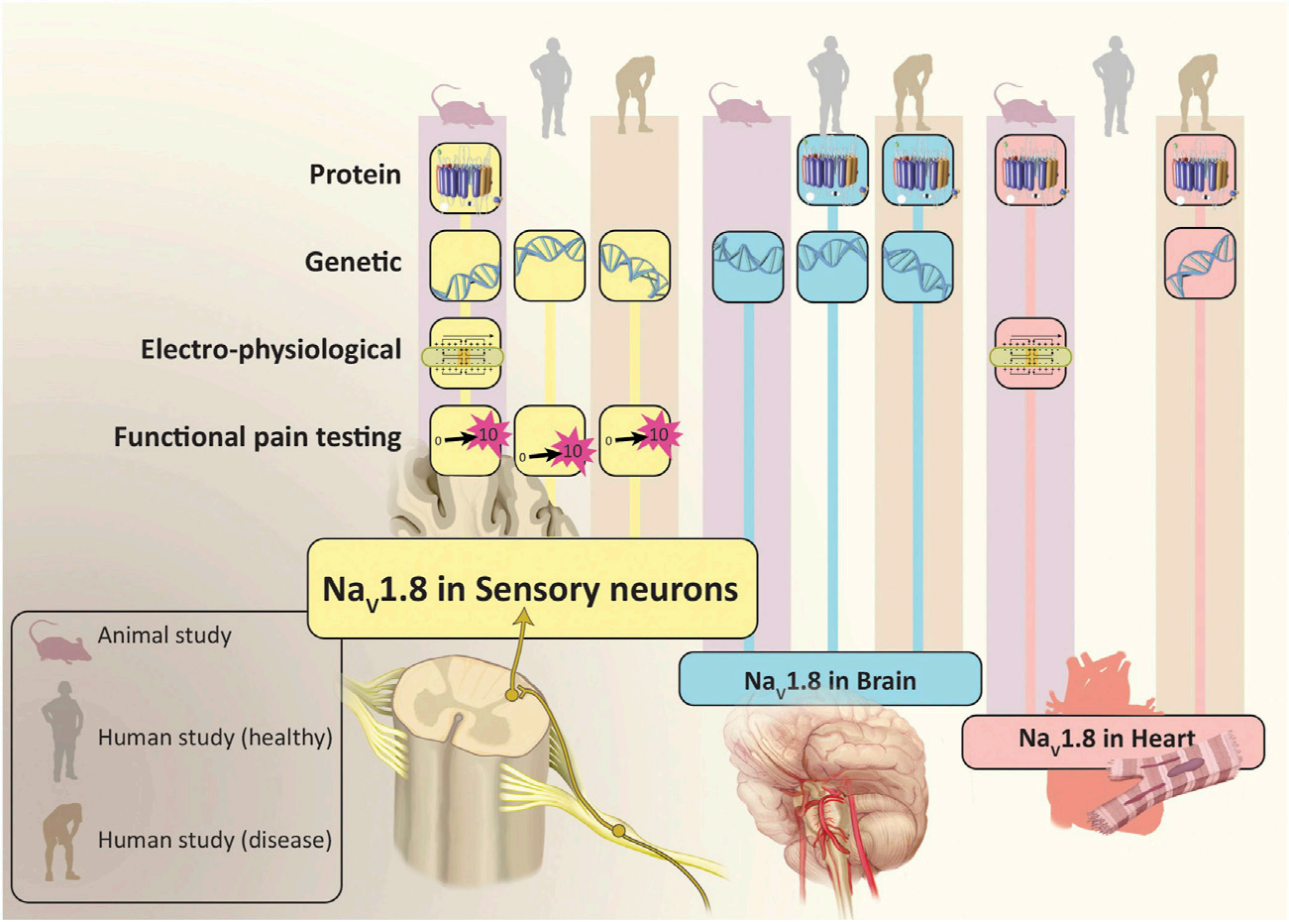
Evidence for Location and Function of Na_V_1.8. In addition to sensory neurons, studies have found that Na_V_1.8 is also expressed in the brain and heart. The population studied (animal, healthy human and human with disease) and evidence which demonstrated Na_V_1.8 (protein, genetic, electro-physiological and functional pain testing) is depicted for each location where Na_V_1.8 has been expressed. Illustration by Devon Stuart.

However, it is important to note that these channels are not exclusively localized to nociceptive neurons. Studies of peripheral nerves in mice have demonstrated that a significant proportion of large fiber, myelinated neurons (indicative of mechanoreceptor function essential for touch sensation) expressed functioning Na_V_1.8 (Shields et al., 2012). Studies performed in mice have also provided immunohistochemical and electrophysiological evidence for the expression of Na_V_1.8 in non-nociceptive neurons within the heart (Verkerk et al., 2012). Genome-wide association studies have found links between specific *SCN10A* polymorphisms and arrythmias or other cardiac disorders in humans. There are also reports of increased Na_V_1.8 mRNA transcript and protein expression in heart tissue obtained from individuals with cardiac hypertrophy and heart failure (Dybkova et al., 2018; Ahmad et al., 2019). Interestingly, no studies to date have confirmed the presence of Na_V_1.8 channels in cardiomyocytes of normal human heart tissue (Casini et al., 2019). Additionally, separate immunohistochemical studies in transgenic mice have identified Na_V_1.8 in brain tissue (Tenza-Ferrer et al., 2022). While Na_V_1.8 expression has not been demonstrated in a healthy human brain, these channels have been identified in Purkinje fibers from the cerebella of rodents used in multiple sclerosis (MS) models, as well as those derived from post-mortem patients previously diagnosed with MS (Black et al., 1999; Black et al., 2000; Waxman, 2005) (Figure 2).

## The role of Na_V_1.8 in visceral pain perception

There are a variety of studies that have directly and indirectly established the importance of Na_V_1.8 to visceral pain perception. *SCN10A* transcript and immunoreactivity for the Na_V_1.8 channel have been demonstrated throughout the gastrointestinal tract, including the stomach, small bowel and colon (Su et al., 1999; Peeters et al., 2006). Electrophysiological currents consistent with Na_V_1.8 have also been identified in DRGs and vagal fibers innervating these organs (Gautron et al., 2011). Additionally, a variety of animal-based studies have shown that systemic and viscerally localized genetic and/or pharmacological antagonism of Na_v_1.8 leads to significant deficits in several visceral pain modalities (Laird et al., 2002; Hillsley et al., 2006; Matthews et al., 2006).

Several animal-based studies have also demonstrated the important role that Na_v_1.8 has in pathological conditions associated with alterations in visceral pain perception. Animal models of enterocolitis have exhibited increases in Na_v_1.8 expression within sensory neurons (King et al., 2009). Previous studies have also suggested that Na_V_1.8 is required for demonstration of spontaneous activity in damaged sensory neurons (Roza et al., 2003) and for generation of enhanced intestinal nociceptor activity in rodent models of colitis (Beyak et al., 2004; Hillsley et al., 2006; Chen et al., 2013). Using a rat model of stress-induced visceral hypersensitivity, Hu et al. reported that affected animals exhibit increased expression of Na_v_1.8 protein and that TTX-resistant sodium channel current density in colonic afferents is increased (Hu et al., 2013). In a separate model of diabetic intestinal neuropathy, they reported that intestinal sensory afferents were hyperexcitable and that Na_v_1.8 expression in dorsal root ganglia was higher than what was exhibited in control animals (Hu et al., 2016). In 2022, Lima et al. investigated the role of Na_v_1.8 in nociception following a surgical incision. Using real time PCR, they demonstrated that Na_v_1.8 mRNA expression increases following surgical incisions in rats, and is reduced with antisense oligonucleotide application (Ma et al., 2008).

In humans, several lines of evidence also support the important impact that *SCN10A* polymorphisms have on pain perception, including that related to the viscera. Duan et al. performed multiple objective pain-related assessments in several hundred study participants who had been genotyped based upon several *SCN10A* gene polymorphisms. They found that homozygosity for one polymorphism (A1073V, encoding an alanine to valine switch at an intracellular loop associated with the channel) was associated with increased thresholds for reporting mechanical pain (Duan et al., 2016). Our study team performed targeted whole exome sequencing in a carefully phenotyped cohort of inflammatory bowel disease (IBD) patients, and found that individuals who were homozygous for A1073V were more likely to exhibit visceral hypoalgesia (or “silent” disease) (Gonzalez-Lopez et al., 2018). In a follow up investigation, we examined a cohort of patients who had undergone a sigmoidectomy and found that individuals who were homozygous for A1073V demonstrated significantly lower post-operative pain scores than those exhibiting the heterozygous or wild-type *SCN10A* genotypes (Coates et al., 2019). We also transfected rat superior cervical ganglion (SCG) with either wild-type or polymorphic cDNA constructs and found that neurons expressing the A1073V variant activated at more depolarized potentials when compared to those with wild type channels, indicating a hypoactive phenotype (Coates et al., 2019). To our knowledge, this is the only Na_v_1.8-associated polymorphism directly linked to a clinical condition associated with altered visceral pain perception that has also had its physiological impact on the channel described (Table 1).

In a study involving several hundred subjects, three separate *SCN10A* polymorphisms (e.g., A2884G, C3218T and T3275C) were associated with reduced risk for the visceral hypersensitivity disorders functional dyspepsia, epigastric pain syndrome and post-prandial distress syndrome (Arisawa et al., 2013). It is not clear what physiological effects each of these variants have on Na_v_1.8, or whether they impact other disorders associated with visceral pain (Table 1).

## Targeting Na_V_1.8 for pain modulation

Considering the unique nature of Na_V_1.8, it is not surprising that it has served as an increasingly popular target for the development of analgesics. In Table 2, we provide an overview of the agents that have been developed for that purpose. In 2007, a small molecule pore blocker, A-803467 was discovered through a trial and error series of experiments that looked to mimic the blocking of TTX-R currents in rat DRG neurons. The authors identified this furanamide molecule and found that it selectively blocks Na_v_1.8 by suppressing spontaneous action potentials *in vitro*. A-803467 has also demonstrated the ability to reduce activity of spinal dorsal horn neurons in animal models of nerve injury (Jarvis et al., 2007). Notably, however, A-803467 exhibited relatively poor oral pharmacokinetics in this study, raising questions about how easy it would be to administer in humans. Separately, Liu et al. provided intraperitoneal injections of A-803467 to Nav1.8 wild type and knockout mice before they received an injection of atropine. The knockout mice and mice that received A-80346 exhibited reduced response to atropine (i.e., reduced rise in heart rate) compared to wild-type mice that did not receive A-803467. These findings suggested that there are potentially concomitant cardiac effects from this agent that might complicate it is application in humans (Liu et al., 2020). In 2010, A-887826, was developed (based upon the design of A-803467), and it demonstrated an ability to inactivate TTX-resistant currents and reduce neuropathic tactical allodynia in rats (Zhang et al., 2010).

**TABLE 2:** Selective Agents for Na_V_1.8. DRG = dorsal root ganglion, TTX = tetrodotoxin, VGSC = voltage-gated sodium channel.

Agent	Mechanism of action	Effects		Notes	References
		Animal studies	Human studies		
A-803467	Na_V_1.8 Antagonist (antidysrhythmic)	Reduced Rat Mechanical Allodynia and Thermal Hyperalgesia	Diminished TTX-R current in human DRG neurons (high dose)		Jarvis et al. (2007)
					Zhang et al. (2017)
A-887826	Na_V_1.8 Antagonist	Reduced antinociceptive activity in rat spinal nerves (tactile allodynia)	Selectively blocked human embryonic kidney (HEK-293) recombinant cells	10-fold more potent in blocking TTX-R Na^+^ compared to A-803467	Zhang et al. (2010)
PF-01247324	Na_V_1.8 Antagonist (antidysrhythmic)	Inhibits TTX-resistant currents *in vivo* rat DRG neurons	Inhibits TTX-resistant current in isolated human low *post mortem* injury L4 and L5 DRG neurons	Orally available	Payne et al. (2015)
					Shields et al. (2015)
PF-04531083	Na_V_1.8 Antagonist		Third molar extraction. Clinical trials have reported little to no effect to date		Clinicaltrials.gov (6 registered trials with NCT numbers)
VX-150	Na_V_1.8 Inhibitor		Analgesic response at up to 10 h in some pain tests (cold pressor most)	Healthy volunteers in Netherlands. Oral	Hijma et al. (2021)
VX-548	Na_V_1.8 Inhibitor		At highest dose (100 mg loading, 50 mg maintenance), reduced acute pain over 48 h	2 phase two trials after abdominoplasty or bunionectomy	Jones et al. (2023)
LTGO-33	Na_V_1.8 Inhibitor	Demonstrated increased efficacy in human DRG when compared to that of dog and rodent	Inhibited Na_V_1.8 in nM range and exhibited 600-fold selectivity compared to other VGSCs	Small molecule inhibitor; equally effective when channel is in activated or closed state	Gilchrist et al. (2024)

In 2015, Payne et al. reported that PF-01247324, another purported selective Na_v_1.8 antagonist, was able to inhibit TTX-resistant currents in isolated human DRG neurons and *in vivo* rat DRG neurons. They also found that this agent exhibited significant selectivity over Na_v_1.5 (another TTX-resistant channel) and TTX-sensitive channels. Of note, unlike studies of A-803467, PF-01247324 reduced nociception in the formalin test. These findings supported the role that Na_v_1.8 has in inflammatory and neuropathic pain (Payne et al., 2015). As described above, previous studies have also reported that Na_v_1.8 may be expressed in the cerebellum during specific disease states (e.g., MS) (Black et al., 1999; Black et al., 2000; Renganathan et al., 2001). Shields et al. demonstrated that PF-01247324 led to diminished cerebellar deficits when administered in mice with autoimmune encephalitis (a model of MS) (Shields et al., 2015). This revealed the potential for Na_V_1.8-directed therapies in MS and other related disorders, but also raised questions regarding its potential to result in centrally-mediated adverse effects. PF-04531083, a compound structurally similar to PF-01247324, has been the subject of a phase 2 clinical trial investigating its impact on pain after dental surgery. Thus far, no significant analgesic efficacy has been reported.

In 2021, a phase one trial was conducted which compared VX-150, an orally bioavailable pro-drug, to placebo in a two-way crossover study to evaluate the analgesic effects of VX-150 in healthy adult males. This was the first human experimental pain study using a selective Na_V_1.8 inhibitor. This study demonstrated that VX-150 influenced cold pressor pain thresholds, with no reported significant adverse effects to patient safety (Hijma et al., 2021). More recently, VX-548 was studied in two phase two trials (evaluating its effects on pain in patients who had undergone bunionectomy or abdominoplasty surgeries) and it was found that, at higher doses, it significantly reduced reported pain scores (Jones et al., 2023). Of note, however, there were multiple reports of headache and constipation. Most recently, Gilchrist et al. reported the development of a new selective small molecule inhibitor of Na_V_1.8, LTGO-33 (Gilchrist et al., 2024). This agent demonstrated high selectivity for Na_V_1.8 when compared to other VGSCs (reportedly 600-fold relative potency). LTGO-33 is also apparently channel state independent. demonstrating similar effects whether the channel is in an inactivated or closed state. Finally, LTGO-33 also demonstrated some degree of species specificity, working most effectively in human dorsal root ganglia when compared to those of other mammalian species.

There is also evidence that other existing medications (including some agents already utilized for their analgesic properties) may target directly or indirectly Na_v_1.8 as well. Tanezumab, a humanized monoclonal anti-NGF antibody, has been shown to reduce expression of Na_v_1.8 positive neurons and result in relative hypoalgesia in animal models, presumably through its effects on inflammatory pathways (Hoffman et al., 2011). Amitriptyline has demonstrated efficacy in reducing chemotherapy-induced neuropathic pain, and patch clamp testing suggests that, when it is applied to isolated DRG neurons, it diminishes activity of Na_v_1.8 (Genevois et al., 2021). The cannabinoid anandamide has been shown to reduce Na_v_1.8 associated currents in cell models (Okura et al., 2014). Ekins et al. used machine learning to generate a list of potential Na_v_1.8 inhibiting compounds from the Prestwick library, which included dihydropyridine calcium channel antagonists (Ekins et al., 2019).

## Current limitations and future directions

Considering the findings shared above, there are many reasons to be hopeful when considering the potential of Nav1.8 to provide new, more effective, and potentially safer methods for managing pain, particularly visceral pain. However, before that full potential is realized, there are still multiple challenges that need to be addressed in order to better understand the physiology of Nav1.8 and to determine how best to target it in this context. For example, none of the agents outlined above have been specifically examined to assess their individual impacts on visceral pain perception. Additional pre-clinical and clinical studies dedicated to evaluating the impact of one or more of these medications on visceral pain perception will be essential in order to determine if they can be used safely and effectively in conditions associated with acute and/or chronic abdominal pain. Pending the outcomes of such studies, further investigation evaluating the optimal drug delivery methods for management of disorders associated with visceral pain may also be necessary (i.e., comparing the efficacy of oral vs. subcutaneous vs. other formulations). This issue would become particularly relevant if there are future attempts to target channels specifically associated with visceral sensory nerves (an important hypothetical to consider if further studies indicate that there are significant adverse effects related to system-wide administration). Beyond this, while several antibodies directed against epitopes on the rodent forms of the Nav1.8 channel exist, the current options result in inconsistent results. There is also a relative paucity of proven antibodies targeting the human channel. Additionally, until recently, there have been relatively few truly exclusive pharmacological agents that only target Nav1.8. This has historically made it challenging to tease out the characteristics that are truly unique to this channel, particularly when considering the other co-habitant TTX-resistant VGSCs, such as Nav1.5 and Nav1.9. Hopefully, the newer agents will help to refine our knowledge in this regard. However, this also highlights the fact that, while advancements in Nav1.8-targeting medications have been made, we are not ready to use any of them in the clinical setting. Finally, another challenge that has complicated our assessment of Nav1.8 in the past has the been the relative lack of specific models that reliably emulate the human channel and its physiology in both health and pathological conditions. Even when evaluating human versions of Nav1.8 channels, if they are not tested in human cells or tissues, significant differences in biophysical assessments may occur. Future testing should incorporate human models (e.g., iPSC-derived or cadaveric neurons) whenever possible to more accurately recapitulate human physiology.

## Conclusion

Many lines of evidence, including from both animal- and human-oriented studies, reinforce the fact that Na_v_1.8 represents a very promising target for management of pain, including visceral pain. Nav1.8 appears to be uniquely situated to help target pain-related signal transmission, due to its relatively focused expression and critical function on dorsal root ganglion neurons. More refined studies are necessary to clarify the exact role and impact that this channel has in relation to pain, particularly in humans. Additionally, further work needs to be done to develop pharmacological agents and techniques that are more selectively targeted for Na_v_1.8. In time, as we improve our ability to manipulate this channel and harness its functionality, we will have an opportunity to provide patients with novel, more potent and safer analgesic options.
